# Explainable AI identifies diagnostic cells of genetic AML subtypes

**DOI:** 10.1371/journal.pdig.0000187

**Published:** 2023-03-15

**Authors:** Matthias Hehr, Ario Sadafi, Christian Matek, Peter Lienemann, Christian Pohlkamp, Torsten Haferlach, Karsten Spiekermann, Carsten Marr

**Affiliations:** 1 Institute of AI for Health, Helmholtz Zentrum München–German Research Center for Environmental Health, Neuherberg, Germany; 2 Institute of Computational Biology, Helmholtz Zentrum München–German Research Center for Environmental Health, Neuherberg, Germany; 3 Laboratory of Leukemia Diagnostics, Department of Medicine III, University Hospital, LMU Munich, Munich, Germany; 4 Computer Aided Medical Procedures, Technical University of Munich, Munich, Germany; 5 Munich Leukemia Laboratory, Munich, Germany; 6 German Cancer Consortium (DKTK), Heidelberg, Germany; 7 German Cancer Research Center (DKFZ), Heidelberg, Germany; Harvard University T H Chan School of Public Health, UNITED STATES

## Abstract

Explainable AI is deemed essential for clinical applications as it allows rationalizing model predictions, helping to build trust between clinicians and automated decision support tools. We developed an inherently explainable AI model for the classification of acute myeloid leukemia subtypes from blood smears and found that high-attention cells identified by the model coincide with those labeled as diagnostically relevant by human experts. Based on over 80,000 single white blood cell images from digitized blood smears of 129 patients diagnosed with one of four WHO-defined genetic AML subtypes and 60 healthy controls, we trained SCEMILA, a single-cell based explainable multiple instance learning algorithm. SCEMILA could perfectly discriminate between AML patients and healthy controls and detected the APL subtype with an F1 score of 0.86±0.05 (mean±s.d., 5-fold cross-validation). Analyzing a novel multi-attention module, we confirmed that our algorithm focused with high concordance on the same AML-specific cells as human experts do. Applied to classify single cells, it is able to highlight subtype specific cells and deconvolve the composition of a patient’s blood smear without the need of single-cell annotation of the training data. Our large AML genetic subtype dataset is publicly available, and an interactive online tool facilitates the exploration of data and predictions. SCEMILA enables a comparison of algorithmic and expert decision criteria and can present a detailed analysis of individual patient data, paving the way to deploy AI in the routine diagnostics for identifying hematopoietic neoplasms.

## Author summary

The analysis of blood and bone marrow smear microscopy by trained human experts remains an essential cornerstone of the diagnostic workup for severe blood diseases, like acute myeloid leukemia. While this step yields insight into a patient’s blood system composition, it is also tedious, time consuming and not standardized. Here, we present SCEMILA, an algorithm trained to distinguish blood smears from healthy stem cell donors and four different types of acute myeloid leukemia. Our algorithm is able to classify a patient’s blood sample based on roughly 400 single cell images, and can highlight cells most relevant to the algorithm. This allows us to cross-check the algorithm’s decision making with human expertise. We show that SCEMILA is able to identify relevant cells for acute myeloid leukemia, and therefore believe that it will contribute towards a future, where machine learning algorithms and human experts collaborate to form a synergy for high-performance blood cancer diagnosis.

## Introduction

Artificial Intelligence (AI) is on the brink of widespread application in healthcare and diagnostics [1]. To a large extent, this rapid development can be attributed to the successful implementation of deep neural networks, unifying feature extraction and classification within one algorithm. While their performance is impressive, it is not per se clear how a classification prediction is made, leading to the term ‘black box models’. For high-stakes decisions however, like a clinical treatment choice, it is imperative that algorithmic decisions are comprehensible and trustable from a human perspective [2].

Extracting the information contained in large image data sets with single cell resolution, deep neural networks have been able to e.g. discriminate cancer types [3], predict cancer patient survival [4,5], classify single blood and bone marrow cells [6,7], and discriminate leukemic subtypes [8–10] with expert accuracy. Towards explainability in a tissue-to-cell level, some approaches [3,11,12] employed attention mechanisms that allow for the identification of relevant regions in gigabyte-large histological scans. While a qualitative agreement between patches deemed relevant by pathologists and the high-attention patches used by the algorithm has been reported [13], a thorough quantitative comparison is missing so far. Post-hoc explainability on the pixel level is designed to highlight relevant image areas and has been provided by a variety of methods, but their usefulness and reliability has been criticized recently [14,15]. Running a traditional feature based approach in parallel to the application of deep neural networks can identify important features [16] and even instruct human experts [17]. However, implementation of explainability methods requires significant extra work, and it is not a given that handcrafted features work as well as the network’s feature extraction. Ideally, an AI algorithm’s decision making is explainable without considering features explicitly, and can be quantitatively compared to expert knowledge.

Here, we showcase the capability of an inherently explainable AI approach on a large dataset of single cell images scanned from blood smears of acute myeloid leukemia (AML) patients. A correct and early identification of AML genetic subtypes is key for successful classification, prognostication, therapy and long-term survival. The most immediate way to identify morphological subtypes is the microscopy-based inspection of a patient’s bone marrow and blood smear. Here, AML is typically detected by identifying more than 20% of all white blood cells (WBCs) as blast cells. Other diagnostic hints are delivered by specific cell anomalies. For example, morphological hallmarks of acute promyelocytic leukemia (APL), an AML subtype with a high risk of potentially lethal bleeding, are atypical promyelocytes and faggot cells, i.e. immature atypical promyelocytes with bundles of large, crystalline cytoplasmic inclusion bodies called Auer rods. Detecting and correctly classifying these cells in the blood smear of a patient is of key therapeutic importance, but can be a challenging needle-in-a-haystack search as these cells generally have low abundances.

Genetic subtype discrimination of AML derived blood smears is an ideal use case for explainable AI for two reasons: First, morpho-genetic correlations between the appearance of atypical cells and the *PML*::*RARA* fusion are established for APL and allow validation of the model. For other subtypes, morpho-genetic correlations are discussed, as in the case of a *NPM1* mutation that is assumed to correlate with the appearance of blasts with a cup-like nuclear shape [18]. Second, the AML genetic subtype is included in the patient information, providing annotated training data without any label noise. Since this data annotation is not on the single cell image level, our approach exploits the machine learning concept of multiple instance learning, where a novel algorithmic module allows the identification of high-attention cells and the comparison with their diagnostic relevance as assessed by human experts.

## Results

### A blood smear cohort comprising 4 AML genetic subtypes, 189 individuals, and over 80,000 single-cell images

**Fig 1:**
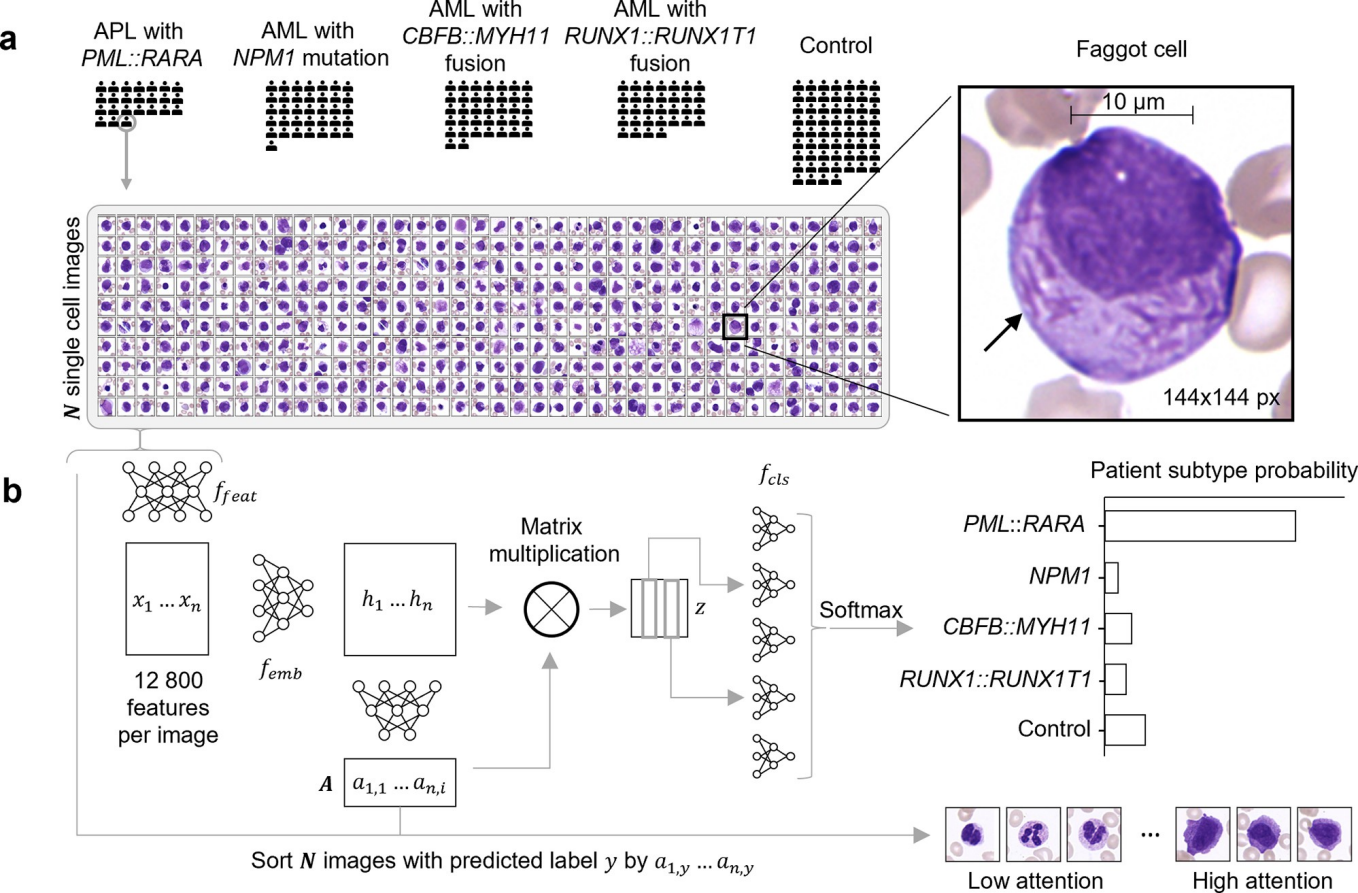
Single-cell based multiple instance learning predicts AML genetic subtypes with high accuracy and identifies clinically relevant cells. (a) Our cohort consists of 81214 single-cell images from 189 individuals comprising four genetic AML subtypes (APL with *PML*::*RARA* fusion (n = 24), AML with *NPM1* mutation (n = 36), AML with *CBFB*::*MYH11* fusion (n = 37), AML with *RUNX1*::*RUNX1T1* fusion (n = 32)) and 60 healthy controls. Single white blood cells were selected and digitized from peripheral blood smears (see Methods). The single-cell images of an exemplary *PML*::*RARA* patient (bottom) contain a *PML*::*RARA* specific faggot cell (right) with clearly visible bundles of Auer rods (arrow). (b) Our single-cell based explainable multiple instance learning algorithm (SCEMILA) classifies patients with an arbitrary number of single-cell images and sorts them by attention. From *N* single-cell images, features are extracted with a pre-trained convolutional neural network *f*_feat_. A bag feature vector is generated via matrix multiplication, which allows patient classification. Moreover, single-cell images can be ordered according to attention, i.e. their importance for a class probability.

The WHO 2022 classification distinguishes AML subtypes according to defining genetic abnormalities [19]. We thus selected the four most prevalent AML subtypes with defining genetic abnormalities and typical morphological features: (i) APL with *PML*::*RARA* fusion, (ii) AML with *NPM1* mutation, (iii) AML with *CBFB*::*MYH11* fusion (without *NPM1* mutation) and (iv) AML with *RUNX1*::*RUNX1T1* fusion. An additional control group of healthy stem cell donors was included. For simplicity, we will refer to the five groups in our cohort as *PML*::*RARA*, *NPM1*, *CBFB*::*MYH11*, *RUNX1*::*RUNX1T1*, and controls. We retrieved 242 blood smears from the Munich Leukemia Laboratory (MLL) database from the years 2009 to 2020 with information on genetics, differential blood counts, patient age and gender (see Methods). Through visual inspection by an expert cytologist (K.S.), we excluded blood smears and single-cell images with insufficient morphologic quality. Additionally, smears with small percentage of diagnostic cells (i.e. if myeloblasts, promyelocytes and myelocytes combined made up for < 20% of leukocytes, as reported in the routine differential blood count) were excluded. After filtering, our dataset comprised 189 blood smears (n = 24, 36, 37, 32, and 60, respectively, Fig 1A) and 81,214 single-cell images, with 430±107 (mean±s.d.) images per individuum. A detailed overview over the filtering steps and how the patient count was affected is given in S1 Fig.

### Single-cell based multiple instance learning classifies AML subtypes accurately

In traditionally supervised deep learning approaches for cytology, single cells have been annotated by experts to generate training data for neural network models [6,9,10,20,21]. Here, we only used patient-level annotation, i.e. the AML genetic subtype, to train SCEMILA (pronounced as ˈsɪmələr), a Single-Cell based Explainable Multiple Instance Learning Algorithm. We extracted 12,800 features from each single-cell image and introduced a novel attention vector to account for the multi-class nature of the problem (see Methods and Fig 1B). This allowed us to assign the most probable AML subtype to each patient and to infer which cells received the highest attention for the prediction of each subtype (Fig 1B).

**Fig 2:**
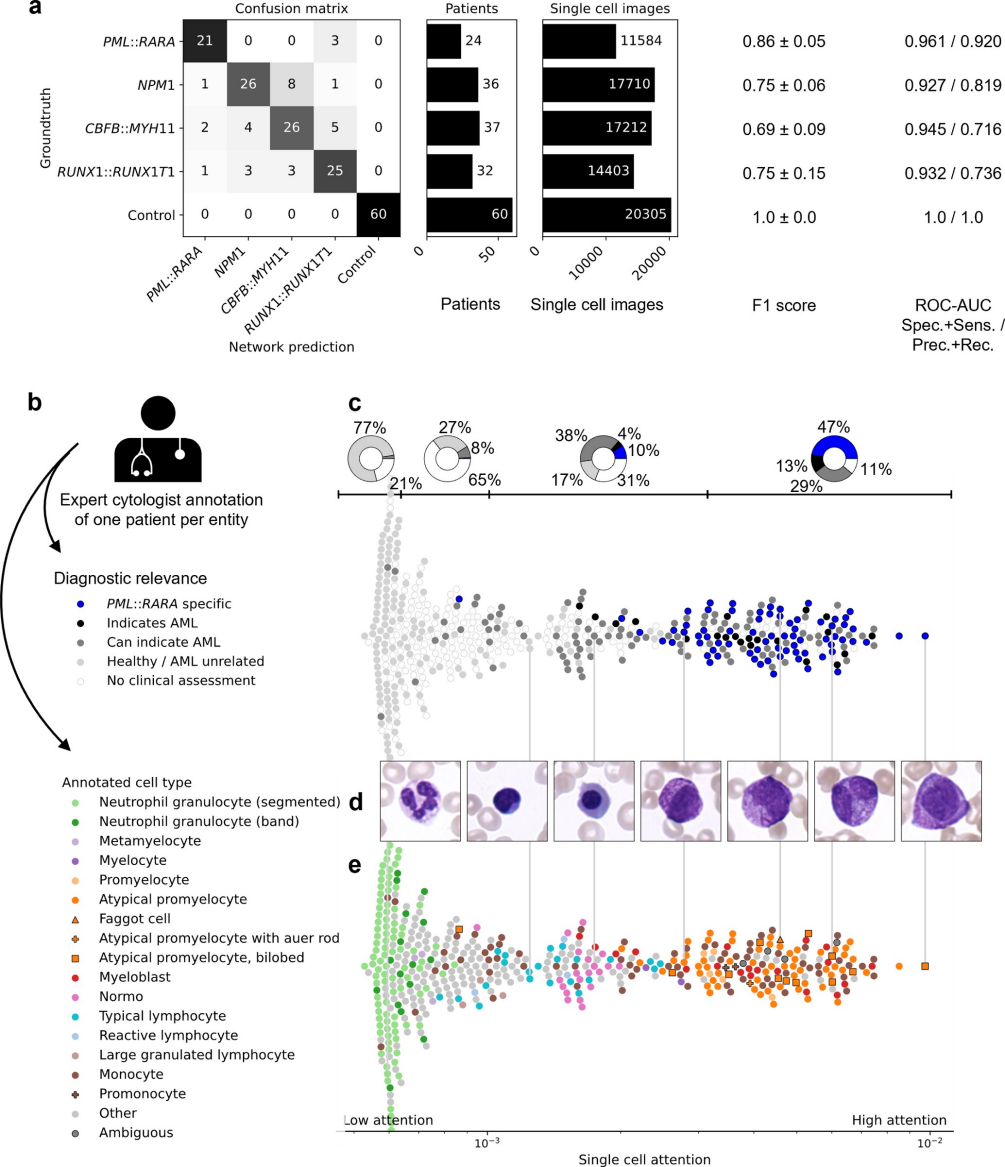
SCEMILA’s single-cell attention coincides with experts’ diagnostic relevance. (a) SCEMILA perfectly differentiated the 129 AML patients from 60 stem cell donors. The cytogenetically different AML genetic subtypes can be distinguished with F1 scores of 0.86±0.05 (*PML*::*RARA*, mean±s.d. from 5 cross validation runs), 0.75±0.06 (*NPM1*), 0.69±0.09 (*CBFB*::*MYH11*), and 0.75±0.15 (*RUNX1*::*RUNX1T1*). Additionally, the ROC-AUC scores for both the specificity/sensitivity (left value) and the precision/recall characteristic (right value) are presented. (b) An expert cytologist annotated one patient per AML entity, assigning diagnostic relevance (Fig 2C) and the morphological cell type (Fig 2E, 2F). (c) In the top attention quartile, 89% of the cells have been annotated diagnostically relevant for AML by an expert hematologist. In contrast, 77% of cells in the low attention quartile have been deemed healthy or AML unrelated. Cells with medium attention ’indicate AML’ or ‘can indicate AML’, according to the expert. Every data point represents a single-cell image from an exemplary, correctly classified *PML*::*RARA* patient. Ticks and pie charts at the top show quartile ranges and diagnostic relevance distribution within quartiles. (d) Individual single-cell images from the *PML*::*RARA* patient show a diverse morphology. The cell with the highest attention (rightmost image) shows strong cytoplasmic granularity and a bilobed, large nucleus. Cells with low attention (leftmost image) show no malignant features. (e) According to expert annotation, atypical promyelocytes (orange) and myeloblasts (red) receive highest attention, while SCEMILA pays little attention to physiological cell types such as neutrophil granulocytes (green), lymphocytes (blue) or other (gray).

We split our data on a patient level into a training, validation and test set and used 5-fold cross validation to estimate split-induced variability (see Methods). SCEMILA was able to perfectly discriminate between healthy controls and AML patients (Fig 2A), and to assign 21 out of 24 patients with *PML*::*RARA* correctly (F1 score = 0.86±0.05, mean±s.d. from n = 5 folds, Fig 2A). High classification performance with F1 scores of 0.75±0.06 (*NPM1*), 0.69±0.09 (*CBFB*::*MYH11*), and 0.75±0.15 (*RUNX1*::*RUNX1T1*) showed that single-cell blood smear morphology can be used to automatically discriminate between AML genetic subtypes, which in turn indicated morpho-genetic correlations learned by our model. For less strict quality criteria for our cohort selection, PML-RARA detection achieved similarly high F1 scores > 0.81 and AUCs >0.90 (S2 Fig).

SCEMILA is able to classify any number of single-cell images provided. We thus used SCEMILA to predict AML subtypes from random patient-wise subsamples of varying size (1 to 500 cells), allowing us to track how model predictions are affected if less data is available. As expected, performance increases for all entities with larger quantities of single cell images, slowly approaching a plateau at around 50 single-cell images (S3 Fig).

### Diagnostically relevant cells achieve high attention

SCEMILA’s novel attention module allows us to order single-cell images according to the attention they received for a specific subtype prediction. To investigate if the model’s attention focused on similar cells as a medical professional would do, an expert hematologist (C.P., see Methods) annotated single-cell images from four correctly predicted patients into 8 diagnostic relevance groups (S1 Table) and 30 different cell types. For an exemplary *PML*::*RARA* patient, model attention coincided with expert hematologist annotation (Fig 2B–2E): Cells that are known to be specific for this genetic subtype (faggot cells and atypical promyelocytes, blue dots in Fig 2C) received the highest attention and constituted 47% of cells in the top attention quartile (Fig 2C). Together with cells that ‘(can) indicate AML’, 89% of all cells in this quartile are deemed diagnostically relevant. In contrast, healthy cells and cells not related to AML received low attention and make up for 77% in the bottom attention quartile. Cells that can indicate AML (or other bone marrow disorders) but are less specific (erythroblasts, monoblasts, monocytes, myeloblasts, myelocytes, normocytes, promonocytes and promonocytes, see S1 Table) received an intermediate level of attention. For an exemplary *NPM1* patient, cells with highest attention were myeloblasts and cup-like blasts (S4A Fig), the latter of which are known to be characteristic, but not specific for AML with mutated *NPM1*. Monocytic cells receive the highest attention and seem to be required by SCEMILA to classify the exemplary *CBFB*::*MYH11* patient correctly (S4B Fig) as expected by the association with myelo-monocytic leukemias [22] and by the absence of pathological eosinophils within the annotated cases (*CBFB*::*MYH11* specific cell type, which is rarely present in peripheral blood but detectable in bone marrow, see S1 Table). For an exemplary patient with a *RUNX1*::*RUNX1T1* fusion gene, all subtype-specific cells appear in the quartile with the highest attention (red dots in upper plot of S4C Fig), as well as almost all of the AML-indicating cells of that patient.

### SCEMILA deconvolves patient blood smear with single-cell resolution

**Fig 3:**
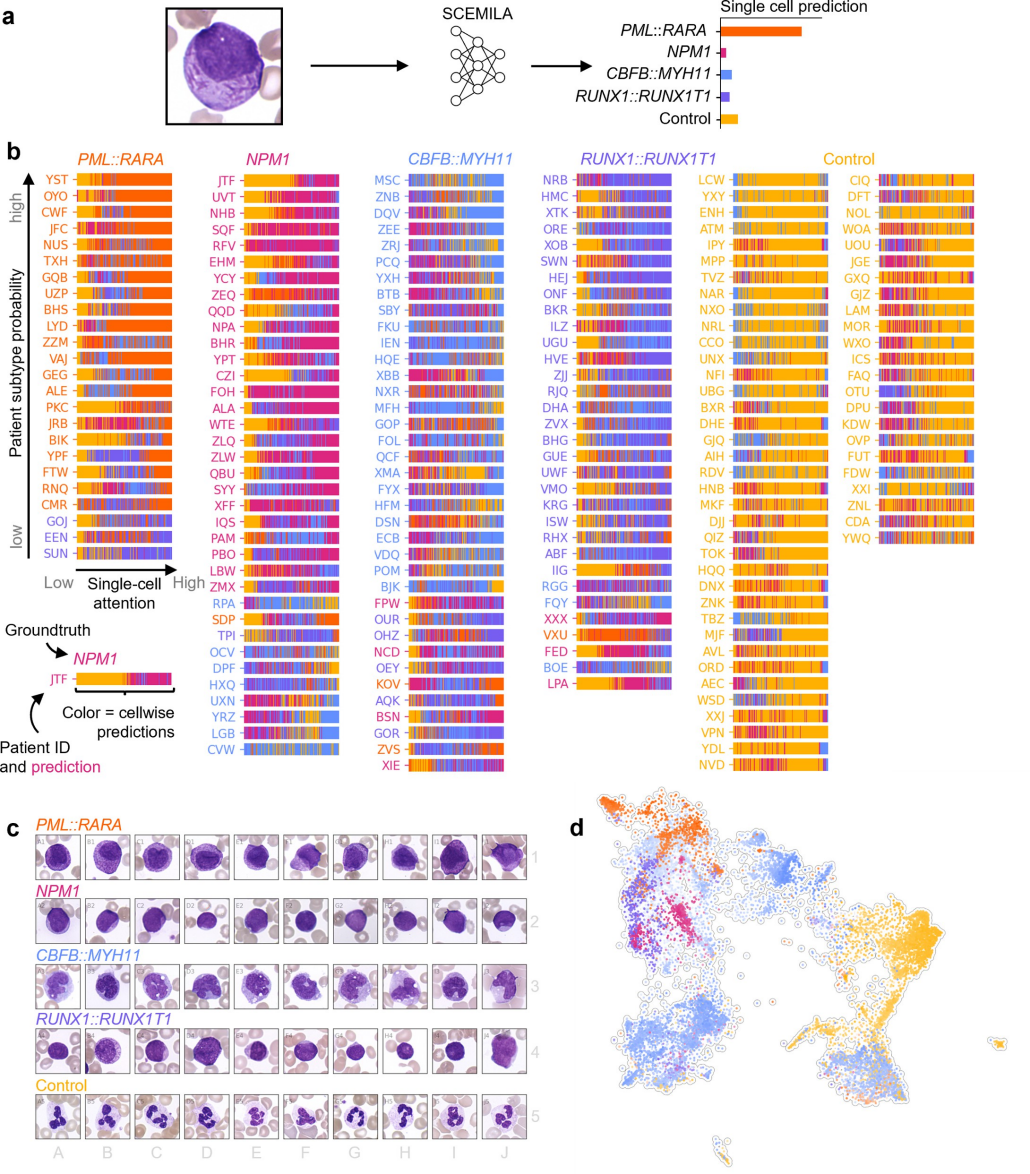
SCEMILA deconvolves patient composition and identifies representative cells for AML subtypes. (a) By passing instances individually through SCEMILA, the algorithm returns single cell predictions. (b) Bar plots show predictions for every single cell of each individual in our dataset. Cells are ordered by attention, with low-attention cells on the left and high-attention cells on the right side. Patients which were classified with strong output activation are presented further up in the list, and the corresponding bag prediction is encoded in the patient label color next to the column. (c) The top 10 cells (2 per fold, based on single cell predictions made by SCEMILA) are displayed for every entity, and single-cell morphology matches with existing medical knowledge. SCEMILA recognizes atypical promyelocytes with strong granulation (*PML*::*RARA*), cup-like blasts (*NPM1*) and myelomonocytic cells (*CBFB*::*MYH11*) as relevant for the respective entities. For our algorithm, smaller blasts are associated with *RUNX1*::*RUNX1T1*, while neutrophil granulocytes are classified as controls. (d) The UMAP embedding shows all cells from the first test set fold. Within the UMAP, the color code represents individual single cell predictions made by our algorithm, with the intensity representing the model output for the respective class. Our approach delineates dedicated morphological cell clusters for every AML entity as well as the controls.

Can we explain SCEMILA’s decision making also for patients without single-cell annotations? To that end, we used the trained SCEMILA model as a single-cell classifier, passing individual cells instead of image stacks to the algorithm (Fig 3A). This allowed us to deconvolve each patient’s blood smear composition without any single-cell annotations. For all 21 correctly classified *PML*::*RARA* patients, high attention cells have all been predicted as *PML*::*RARA* (Fig 3B). The three misclassified *PML*::*RARA* patients are all predicted as *RUNX1*::*RUNX1T1* due to the cells with the highest attention. According to expert hematologist assessment (K.S., see Methods), two of these (GOJ and EEN) show granulated blasts suspicious for APL from manual inspection (see S5A, S5B Fig). For the third misclassified patient (SUN) few blasts are present and the frequency of neutrophils is decreased (S5C Fig), but no AML diagnosis would be inferred from the single-cell images alone in a clinical setting. Notably, the algorithm assigns high attention to suspicious AML cells, but does not predict the proper subtype. Correctly classified *NPM1* and *RUNX1*::*RUNX1T1* patients present with high attention for subtype specific and AML-indicating cells (S4A, S4C Fig). For *CBFB*::*MYH11* the picture is more mixed (S4B Fig), while for control individuals, *NPM1* classified cells often received high attention, but correct bag classification is apparently dominated by the large amounts of single cells classified as control.

### Identification of subtype specific single-cell features

By ranking cells based on the model output for single-cell classification(see Methods), we were able to highlight subtype specific morphological features (Fig 3C). Cells classified as *PML*::*RARA* with the strongest probability were mostly large, mostly granulated promyelocytes and myeloblasts. Some of them were bilobed (Fig 3C, cell C1), which matches well with known distinctive features of *PML*::*RARA*. *NPM1* cells were mostly rather small, with comparatively little cytoplasm and an incidental cup-like morphology (Fig 3C, cell D2, J2). *CBFB*::*MYH11* cells presented myelomonocytic features (e.g. Fig 3C, cell A3, E3, G3), while *RUNX1*::*RUNX1T1* showed the smallest cells with the least amount of cytoplasm (cells A4 and F4), and occasional larger myeloid progenitor cells (cells B4, D4). As expected, control cells show healthy neutrophil granulocytes in accordance with clinical knowledge (cells A5-J5).

### Single-cell UMAP embedding

We mapped single cells into a UMAP [23] embedding that also contains 1,983 expert annotated single-cells (see Fig 2E) serving as landmarks for lymphocytes, myeloid progenitors, monocytes, neutrophils, eosinophils, and debris (see S6 Fig). By visualizing the output activations for individual cells, SCEMILA revealed AML subtype-specific cell clusters (Fig 3D): While *PML*::*RARA* cells (orange) formed two isolated promyeloid clusters, two types of *NPM1* blasts appeared (red) that partly overlapped with *RUNX1*::*RUNX1T1* blasts (blue). Cells predicted to be *CBFB*::*MYH11* populated a confined region associated with monocytes and monoblasts, but also, surprisingly, the lymphocyte region of the UMAP. The neutrophils of the control class (green) separated nicely in the cluster of mature healthy cells. A full interactive exploration of single cell attentions, patient wise UMAP embeddings, single cell images and model predictions is possible via our interactive online tool (https://swarmplot.helmholtz-muenchen.de/pkc_visualization.html).

## Discussion

Computational blood smear analysis offers the unique opportunity to relate important model features to cytological expertise, accumulated over decades of clinical research and practice. In contrast to previous approaches[8,24,25], SCEMILA weighs individual images and can thus focus even on few, diagnostic cells. This procedure mimics the approach to blood smear evaluation taken by human experts, who sometimes conclude solely based on a few pathognomonic cells. Moreover, it allows for the quantification of congruence of AI attention and expert’s diagnostic relevance, and thus provides explainability.

In routine diagnostics, SCEMILA has the potential to support the clinical workflow by highlighting rare but diagnostically relevant cells, allowing cytologists to accelerate tedious cell-by-cell blood smear microscopy and focus on important cells right away. By deconvolving a patient’s single-cell composition and embedding images into a low-dimensional map, the morphologic diversity of leukocytes present in a blood smear is summarized in an easy-to-read visualization. To that end, our interactive online maps provide a quick impression of myeloblast frequency and morphology and can highlight individual cells with high attention. Instead of identifying, classifying and counting single leukocytes, cytologists can use these maps to query suspicious single cells in the context of the whole slide, and scrutinize a suggested disease classification. For successful implementation of SCEMILA into a cytologist’s daily routine however, standardized smear preparation and scanning as well as a seamless integration of the algorithm into the digital lab workflow have to be established.

The diagnostic situation for which a model is trained is a design choice that determines its future applicability. We here focused on four distinct WHO defined genetic subtypes of AML. Accordingly, our trained model can only be used to differentiate these specific subtypes, as the learned correlations do not generalize per se to a more diverse set of entities. SCEMILA shares this limitation with recent publications in computational pathology that used tissue-level morphological data to predict genetic properties [26,27]. Generalization has proven difficult especially for blood and bone marrow smear microscopy, as handling, staining and scanning all are highly variable and so far poorly standardized across different laboratories [7]. Consequently, while an implementation of SCEMILA for bone marrow samples is desirable and can yield additional clinical benefit, our study focused on processing regular blood smears, which are much easier to obtain, stain and digitize. A follow-up analysis with a larger cohort and a broader spectrum of genetic alterations as well as an extension to bone marrow smears will be required to test the generalisability of approaches like ours and evaluate whether SCEMILA can identify frequent AML mutations in a clinical scenario. Extending the number of subtypes during training might also help to discriminate class features in the learned embedding in a more specific manner. Our observation that *CBFB*::*MYH11* patients are classified partly due to myelomonocytic, but partly also due to high-attention lymphocytes hints towards room for improvement with respect to class-specific features. Methodologically, it will be interesting to see how the application of transformers, machine learning methods that include the concept of attention naturally in their architecture, perform on cytological data. With an algorithm that can highlight individual cells, a promising direction for further studies will be the development of approaches for early relapse detection, where small cell populations can have strong diagnostic impact. However, the dataset and algorithm provided in this paper will need to be supplemented and fine-tuned with frequent longitudinal patient samples and experiments to evaluate such a clinical use case.

In terms of explainable AI, robust and reliable methods on the pixel level are still missing. They have to be established, tested, and merged with the concept of multiple instance learning to improve explainability further, with the potential to extract novel morphological features with diagnostic value. Leveraging other data modalities, like clinical data and electronic health records offers further potential to boost performance, but also to explain the importance of features between modalities. As with all medical machine learning research, prospective trials form an indispensable step to ensure high medical standards [28–30] and are already being conducted for single white blood cell classification [31]. It will be interesting to see how algorithms perform on real world data, e.g. the unfiltered input of a medical laboratory. Here, explainability is key to make decisions transparent (in particular in difficult cases), help find rare cells, and quickly identify prediction errors. Our algorithm is a first step towards the future of explainable AML assessment, where human expertise is combined with machine learning to form a powerful synergy for efficient and standardized disease classification.

## Materials and methods

### Data

#### Ethics

The Munich Leukemia Laboratory (MLL) diagnoses a broad spectrum of hematologic diseases and preserves sample material used for research studies if written patient consent is given. All patients included in this study gave consent and were > 18 years old. Our analysis was approved by the medical faculties’ ethics committee of the Ludwig-Maximilians-University (proposal IDs: 19–969 and 21–0284).

#### Cohort selection

242 samples were selected from the MLL blood smear archives based on final diagnosis. We focused on four AML subtypes that emerge from genetic mutations and alterations: APL with *PML*::*RARA* fusion (n = 51), AML with *NPM1* mutation (n = 45), AML with *CBFB*::*MYH11* fusion (without *NPM1* mutation) (n = 47), and AML with *RUNX1*::*RUNX1T1* fusion (n = 38). As covering the entire molecular spectrum of AML within a single dataset is almost impossible due to strong class imbalance, these classes were selected as they comprise more than half of all AML cases in patients up to the age of 65 years [32]. An additional stem cell donor cohort (n = 61) was acquired to provide healthy controls. Every sample was processed and archived between 2009 and 2020. Due to strongly imbalanced AML subtype frequency, samples are not matched in time.

#### Scanning

In a first step, the blood smear is scanned with a 10x objective. An overview image is created from the individual images. Cell detection is performed in each image field by the *Metasystems Metafer* software. After applying a segmentation threshold and a logarithmic color transformation, specifically stained cells with an object size between 40–800 μm^2^ are detected and stored in a gallery image of 144x144 pixels. Each gallery image is then assigned to a quality level using a DNN that determines the region density and further analysis of the immediate cell neighborhood. A 40x position list is then calculated from the cells with high quality in such a way that the largest possible number of leukocytes with sufficient quality are positioned in each image field. Cell detection in the 40x scan is performed in the same way as in the 10x scan using a segmentation threshold, a logarithmic color transformation and an object size between 40–800 μm^2^.

Single-cell images were stored on a hard drive in TIF format with a size of 144x144 pixels, corresponding to 24,9μm x 24,9μm (Fig 1A). By scanning 242 blood smears, we generated a dataset with a total 101,947 single-cell images, with 99 to 500 images per patient.

#### Data cleaning

In clinical routine a leukemia diagnosis is usually derived from multiple sources, while SCEMILA focuses on blood smears only. To address this limitation provide high quality data, we filtered our dataset:

- Blurry images were excluded on a single image basis. Using python and the openCV library, canny edge detection was applied. This function detects orientation and gradient of edges which results in less or no edges should the image be blurred. We filtered out single-cell images where the sum of all edges over the entire image was < 5x10^4^.
- AML samples where myeloblasts, promyelocytes and myelocytes combined made up for < 20% of all images were excluded. This percentage was derived from routine laboratory differential blood counts based on a different set of cells than the single-cell images used for training our algorithm. The 20% threshold represents the required blast percentage for most AML subtypes according to the WHO [22]. Of note, diagnosis can be inferred with lower blast counts in a clinical setting for some subtypes through the presence of pathognomonic cells. However, the 20% threshold was applied to all AML classes to avoid that the algorithm could recognize a specific entity simply by detecting suspicious cells in a quantitative, subtype-specific range.
- Sub-samples of 96 cells per patient were assessed by an expert hematologist (K.S., >10 years of expertise in hematological cytomorphology) to exclude data artifacts such as poor selection of the scanning area, sample degradation and insufficient staining. Patients were excluded if < 25% of single cells would be assessed in a clinical setting, and/or no pathological cells were present despite the myeloblast filtering step.

Filtering our dataset resulted in 189 patients and 81,214 single-cell images. Those were used for all later analysis. Notably, a range of artifacts such as autolytic cells and erroneously digitized red blood cells or platelets still remained in the dataset, increasing the task complexity. For further information, see S1 Fig.

### Single-cell feature extraction

A white blood cell dataset with over 300,000 annotated single-cell images from 2205 blood smears was used to train a ResNet34 [33] model *f*_*feat*_ for single-cell feature extraction. White blood cell images were annotated into 23 individual single-cell classes by experienced MLL cytologists[20]. Cells annotated as erythroblast (19 images) and proerythroblast (2 images) were excluded due to class size. A list of classes *C*_*sc*_ with size and classification performance of the best performing fold (5-fold cross validation with 60-20-20 split) is presented in S7 Fig. Single-cell images were augmented by random horizontal/vertical flipping, rotation, translation, rescaling and random erasing of small areas [34,35] (for implementation details, see code). Probabilistic oversampling was applied to address class imbalance. The model was initialized with weights trained on ImageNet [36] and optimized using categorical cross-entropy: for every single-cell image *I*_*i*_ having an associated label ${l_{i}}^{sc}\in C_{sc}$, training loss for feature extraction step *L*_*feat*_ can be defined as

$$L_{feat}(\theta )=CCE({l_{i}}^{sc},{{\hat{l}}_{i}}^{sc})$$

where ${{\hat{l}}_{i}}^{sc},x_{i}=f_{feat}({I_{i}}^{};\theta )$. *C*_*sc*_ is the set of all classes in the single-cell dataset, ${{\hat{l}}_{i}}^{sc}$ is the estimated class and *θ* is the set of learnable parameters of the model. A feature vector *x*_*i*_∈ℝ^*d*^ with *d* = 12800 represents the flattened activations of the 34th layer of the model before the final 2D average pooling and fully connected layers, which we extracted as the feature vector associated with the input image.

After each epoch, the model was evaluated on the validation set and training continued until no further improvement in validation loss was observed for 10 consecutive epochs. In our case, validation loss reached the highest value after 51 epochs with stochastic gradient descent and a learning rate of 5x10^-4^. Among the different folds of cross-validation, the model with the best performance on the test set was selected to perform feature extraction in our separately scanned multiple instance learning dataset.

### Attention-based multiple instance learning

Multiple instance learning [37] (MIL) allows training a model when labels on a bag level (here: the patient diagnosis), but not an instance level (the single-cell images) are available. Attention-based MIL [38] is an approach where MIL is combined with a trainable attention module weighting the instances without compromising performance of bag-level prediction. This allows the algorithm to predict the label of a bag (here: the AML genetic subtype) by only considering specific instances within this bag (here: selected single-cell images from one patient).

Specifically, we are proposing a permutation invariant method *f*(.) to analyze a set of single-cell images *B* belonging to a patient to return the associated AML subtype *y*∈{*PML*::*RARA*, *NPM1*, *CBFB*::*MYH11*, *RUNX1*::*RUNX1T1*, *control*}. A set of attention scores *α*, showing the importance of every cell in the bag for the classification of the different bag labels, is returned to support the model’s decision:

y,α=f(B)

We designed an attention-based MIL model as follows:

$$L_{MIL}(\phi ,\gamma ,\varphi ,\rho )=CCE(y,\hat{y})$$

where *y* = *f*_*MIL*_({*h*_1_,…,*h*_*N*_}, *A*; *ϕ*), *N* is the number of instances in the bag, *A* is the set of attention scores calculated for the bag and *h*_*i*_ = *f*_*emb*_(*x*_*i*_; *γ*) are the embedded feature vectors obtained by further analysis of the initial feature vectors *x*_*i*_. $\hat{y}$ is the true label, *ϕ* and *γ* are the learnable parameters of the respective steps. Our novel class-wise attention matrix is calculated as

$$\alpha_{i,k}=\frac{exp\{W^{T}tanh(V_{i}{h^{T}}_{k})\}}{\sum_{j=1}^{N}exp\{{W_{i}}^{T}tanh(V_{i}h_{k})\}},\forall c_{i}\in C$$

where *α*_*i*,*k*_∈*A* is the attention score of the instance *k* for class *i*. {*W*, *V*}∈*φ* are learnable parameters obtained by training. Based on the attention matrix, our attention based MIL pooling is done by:

$$z=\sum_{k=1}^{N}A_{i,k}h_{k},\forall c_{i}\in C$$

where z are the bag features which are further processed to obtain the final multi-class prediction *y* = *f*_*cls*_(*z*; *ρ*) and its corresponding attention scores *α* = *A*_*y*_.

This entire process of class-wise attention estimation was designed to eliminate the interclass competition of attention values. A matrix consisting of multiple attention values per instance is generated so that the different attention values only directly influence the prediction of their corresponding class.

Our attention-based multiple instance learning algorithm was implemented in Pytorch and is available at https://github.com/marrlab/SCEMILA.

### Training

Our algorithm was initialized randomly and trained using 5-fold cross-validation: 60% of the samples for each class were used for optimizing parameters, until loss reached a minimum for inference on the validation set (20%). Afterwards, performance for each fold was assessed on the remaining 20% (testing set) and all folds from cross-validation were pooled to obtain our final confusion matrix containing every patient once (Fig 2A). The order of single-cell images within the bag was permuted during training. We used a learning rate of 5x10^-5^ and stopped training after no improvement in validation loss could be seen for 20 epochs, allowing a maximum of 150 epochs. For the 5 folds, the last improvement was observed after 50–75 epochs. As single-cell image feature extraction was done in a separate first step, training time was very short with approximately 15 minutes per fold when trained on an Nvidia Tesla V100 graphics card.

### Follow-up annotations

To correlate the algorithm’s single-cell attention with diagnostic relevance, an expert hematologist (C.P., >10 years of expertise in hematological cytomorphology) annotated all single-cell images from one patient from each subtype. Patients selected were morphologically diverse, had good sample quality and were predicted correctly. We used an in-house tool for online annotation of 1983 cells according to the scheme presented in S1 Table. Cells were pseudonymized and shuffled, depriving the expert from further information and mimicking the conditions for our algorithm.

### UMAP

We used single-cell images from one fold of our dataset to construct a low dimensional embedding using UMAP [23,39] from 12,800 features per image retrieved with single-cell feature extraction. Cells derived from further patients not contained in the initial embedding were then mapped into the 2-dimensional space (Fig 3D, S6 Fig).

### Single-cell classification

SCEMILA’s architecture allowed us to classify individual cells from a patient as well as a whole patient, generating single-cell AML subtype predictions. We were thus able to explain which cell types were used to classify patients into the existing AML categories. For every fold from our 5-fold cross-validation, all single-cell images of the respective test set were passed through the algorithm individually, and single-cell predictions were calculated (Fig 3A). All single-cell predictions are displayed in Fig 3B for every individual patient, with misclassifications indicated by the patient label color. By sorting cells according to their output activations for the different bag labels (AML entities or control), the top 2 cells for every entity and fold are displayed in Fig 3C as a representative example of the subtype specific morphological features. To highlight single cells with their single-cell classification within the UMAP, we show all single cells from one iteration of cross-validation, encoding the predicted label with the color, and the predicted probability with the intensity of the respective datapoint (Fig 3D).

## Code availability

All code used for the project is available at https://github.com/marrlab/SCEMILA.

## Supporting information

S1 Table Single-cell annotation and diagnostic relevance scheme. (XLSX)

S1 Fig Consort diagram. (a) Consort-like diagram depicting our data processing and experimental design. First, individual blurry images were excluded from multiple patients, then entire patients were filtered by manual slide quality assessment and based on results from the routine differential blood count. Afterwards, we split the remaining patients using 5-fold cross-validation, and trained 5 different SCEMILA models. (b) 32 exemplary single-cell images excluded by our canny edge detection filter.
(TIF)

S2 Fig Extended performance metrics for different filter criteria applied to the dataset. To evaluate our algorithm under different circumstances, we evaluated different dataset compositions by applying different filter criteria. Next to the distribution of pathological cells (a) within samples of our dataset (sum of myeloblasts, promyelocytes and myelocytes), performance metrics (precision, recall, F1-measure, sensitivity and specificity) as well as the corresponding confusion matrices are presented, depending on whether we filter (b) only samples with insufficient quality as assessed by a trained expert, (c) additionally exclude samples with no pathological cells according to human cytologist annotation or (d) also exclude all samples with less than 20% pathological cells, as presented in main Fig 1. (e)—(i) show the corresponding ROC curves for all 5 classes for both the sensitivity/specificity and precision/recall characteristic for all 3 filtering scenarios, and the corresponding AUC values are shown within the plots.
(TIF)

S3 Fig Prediction accuracy plateaus already with around 50 single-cell images. (a) Per patient, 100 randomly subsampled single-cell image sets of different size (1, 2, 5, …, max) of the test set were evaluated. SCEMILA’s mean classification accuracy over all patients from the entire dataset plateaus at 50 images. The data point for a “random cell from dataset” were calculated by randomly sampling cells from our entire dataset (regardless of patient groundtruth). (b)-(f) Mean of the output activations for the groundtruth class as generated by SCEMILA for 100 random subsamples. Individual patients are displayed as gray lines, the black line shows the average over all patients from the respective entity.
(TIF)

S4 Fig Attention distribution for three exemplarily annotated patients. Patients with (a) NPM1, (b) CBFB::MYH11 and (c) RUNX1::RUNX1T1 are classified according to myeloblasts (for CBFB::MYH11: monocytes) present in their corresponding smear. Interestingly, the classification for CBFB::MYH11 (b) mainly focuses on monocytic cells to discriminate this subtype from the other types of AML, while classical myeloblasts receive low attention. Ticks and pie charts at the top indicate quartile ranges and cell group distribution within quartiles.
(TIF)

S5 Fig The three misclassified *PML*::*RARA* cases. Out of 24 *PML*::*RARA* cases in our dataset, 3 cases have been misclassified by SCEMILA (see Fig 2A and Fig 3B) as *RUNX1*::*RUNX1T1*. We show 96 representative single-cell images ordered by decreasing attention and the output activation of SCIMILA. (a) Patient EEN contains many white blood cells without intact cytoplasm. Some cells present a bilobed nucleus or stronger granulation. (b) Patient GOJ shows large cells with cytoplasmic granulation as well as some Auer rods. Yet, this patient presents with many artifacts and a lot of red blood cells, some images even contain no white blood cells at all (bottom). Overall the algorithm shows activation for *PML*::*RARA*, *CBFB*::*MYH11* and *RUNX1*::*RUNX1T1*, indicating uncertainty of the classification. (c) While the fraction of neutrophil granulocytes is quite small, patient SUN presents with few suspicious *PML-RARA* cells.
(TIF)

S6 Fig Human annotation provides landmarks within UMAP embedding. All single-cell images from one fold were embedded based on extracted features, using the uniform manifold approximation and projection method (UMAP), and are abstracted by a gray contour. 1983 cells from 4 patients, annotated by an expert hematologist after training, are highlighted, including cells specific for different genetic subtypes of AML. Images show exemplary single cells, clusters for debris (gray), neutrophil granulocytes (green), myeloblasts (red) and (atypical) promyelocytes (orange), lymphocytes (blue), monocytes (brown) were manually annotated. The black arrow highlights the differentiation trajectory from myeloblasts over promyelocytes, myelocytes, metamyelocytes and band neutrophil granulocytes to segmented neutrophil granulocytes.
(TIF)

S7 Fig Confusion matrix for best performing fold of the single-cell classifier used for feature extraction. (TIF)
