# Supplementary figures and images for: Explainable AI identifies diagnostic cells of genetic AML subtypes
Source: PLOS Digit Health. 2023 Mar 15;2(3):e0000187. doi: 10.1371/journal.pdig.0000187 (PMC10016704; doi:10.1371/journal.pdig.0000187)

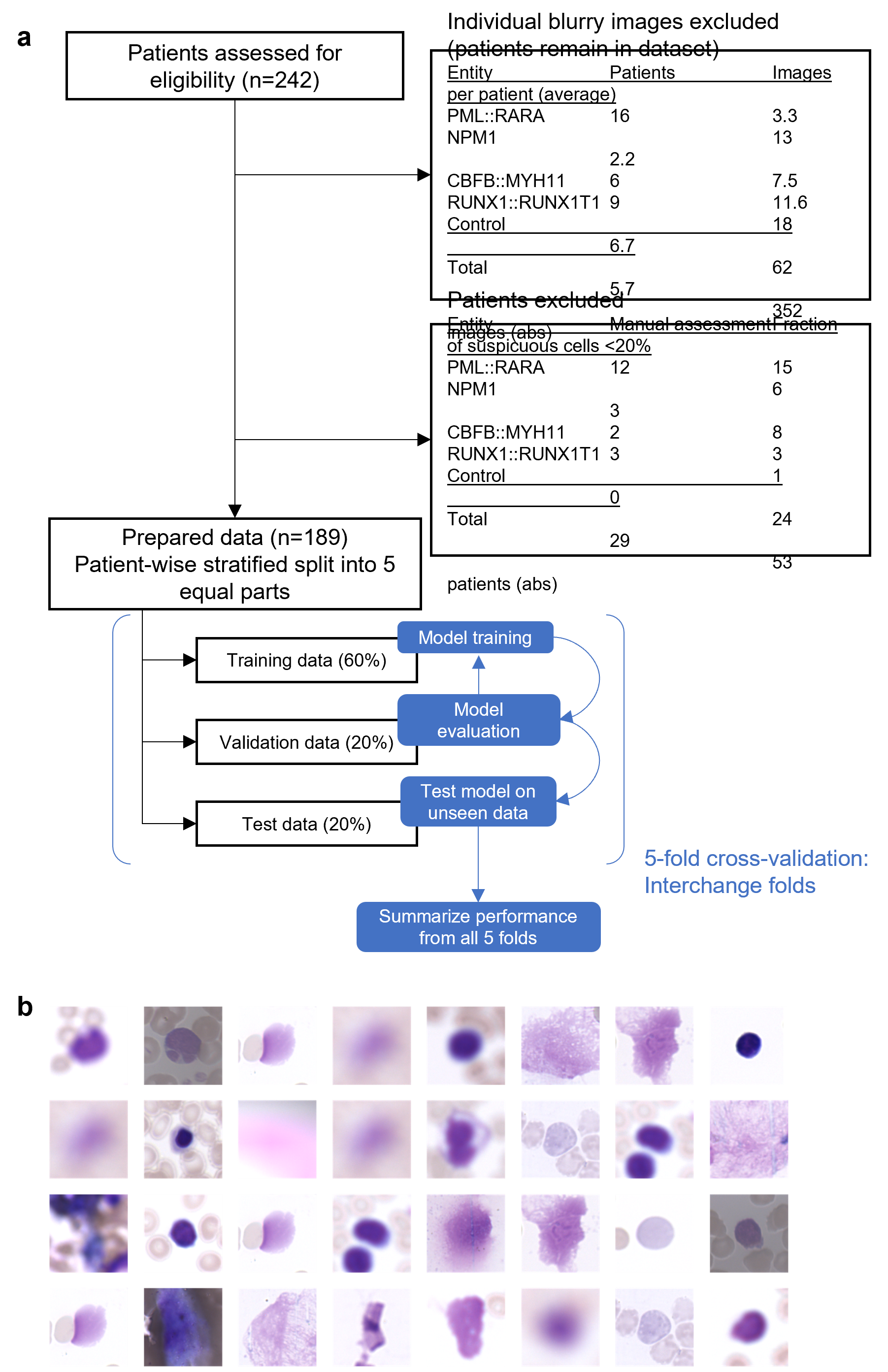

Supplement: S1 Fig — (a) Consort-like diagram depicting our data processing and experimental design. First, individual blurry images were excluded from multiple patients, then entire patients were filtered by manual slide quality assessment and based on results from the routine differential blood count. Afterwards, we split the remaining patients using 5-fold cross-validation, and trained 5 different SCEMILA models. (b) 32 exemplary single-cell images excluded by our canny edge detection filter. (TIF) [file pdig.0000187.s002.tif]

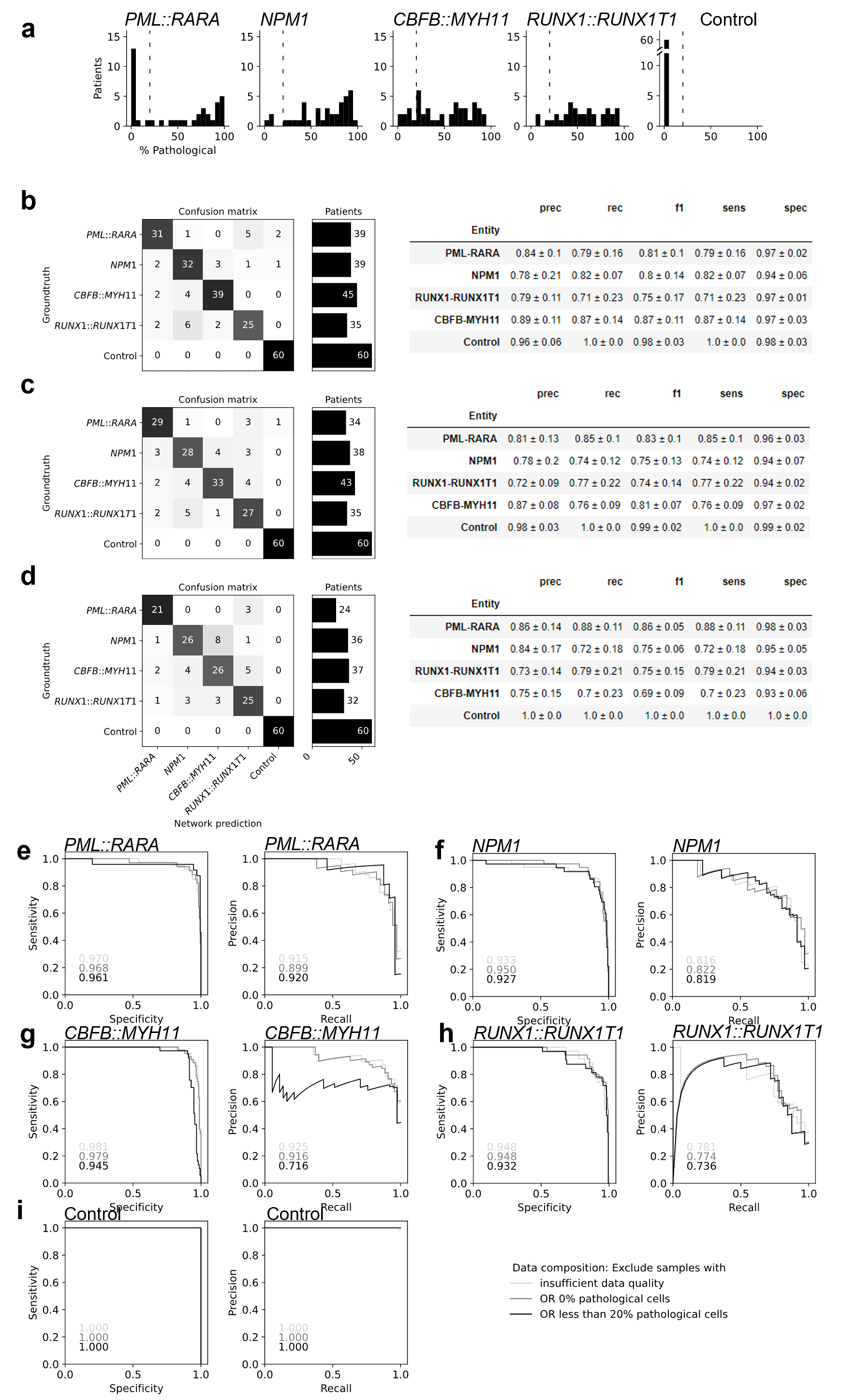

Supplement: S2 Fig — To evaluate our algorithm under different circumstances, we evaluated different dataset compositions by applying different filter criteria. Next to the distribution of pathological cells (a) within samples of our dataset (sum of myeloblasts, promyelocytes and myelocytes), performance metrics (precision, recall, F1-measure, sensitivity and specificity) as well as the corresponding confusion matrices are presented, depending on whether we filter (b) only samples with insufficient quality as assessed by a trained expert, (c) additionally exclude samples with no pathological cells according to human cytologist annotation or (d) also exclude all samples with less than 20% pathological cells, as presented in main Fig 1. (e)—(i) show the corresponding ROC curves for all 5 classes for both the sensitivity/specificity and precision/recall characteristic for all 3 filtering scenarios, and the corresponding AUC values are shown within the plots. (TIF) [file pdig.0000187.s003.tif]

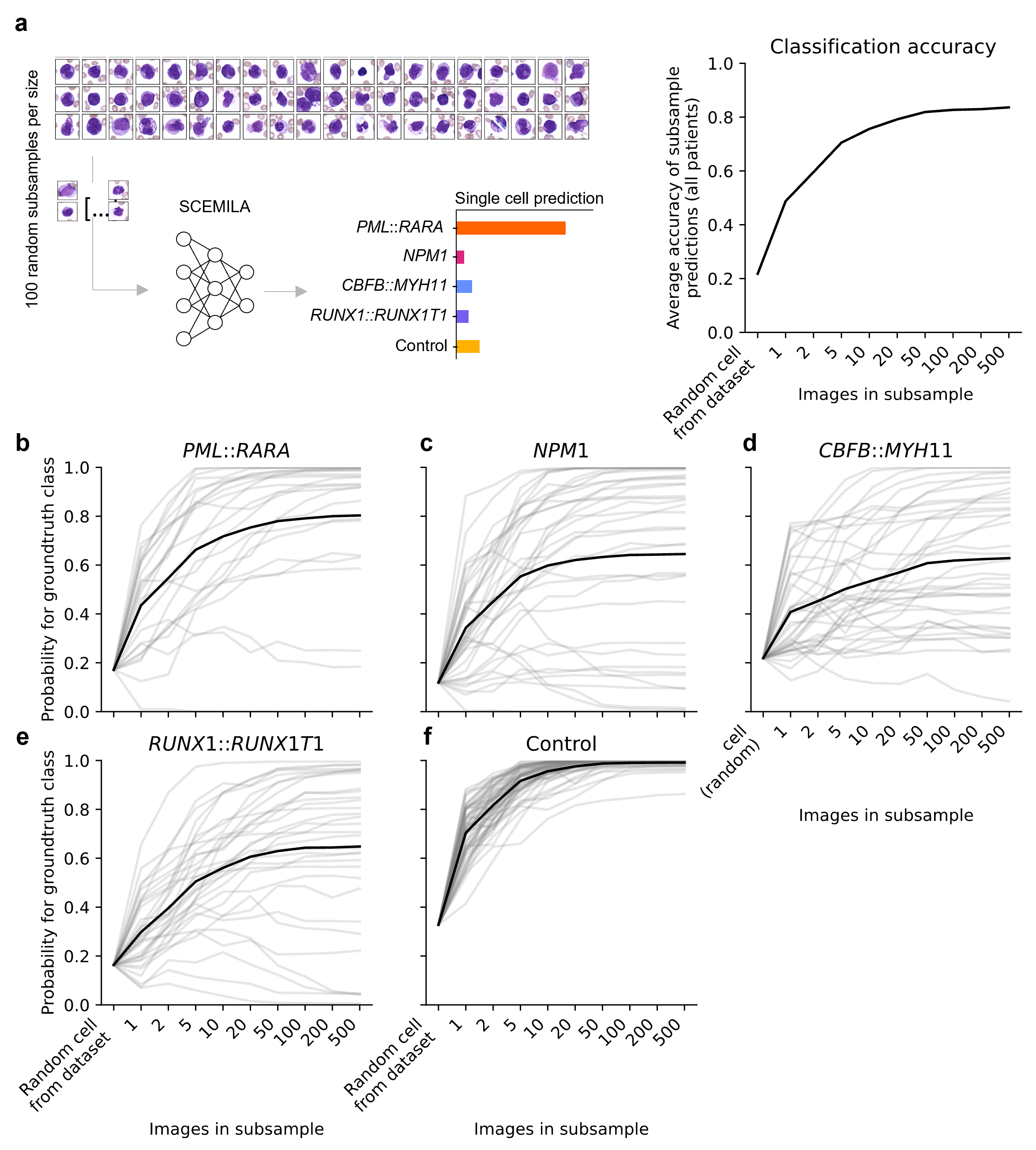

Supplement: S3 Fig — (a) Per patient, 100 randomly subsampled single-cell image sets of different size (1, 2, 5, …, max) of the test set were evaluated. SCEMILA’s mean classification accuracy over all patients from the entire dataset plateaus at 50 images. The data point for a “random cell from dataset” were calculated by randomly sampling cells from our entire dataset (regardless of patient groundtruth). (b)-(f) Mean of the output activations for the groundtruth class as generated by SCEMILA for 100 random subsamples. Individual patients are displayed as gray lines, the black line shows the average over all patients from the respective entity. (TIF) [file pdig.0000187.s004.tif]

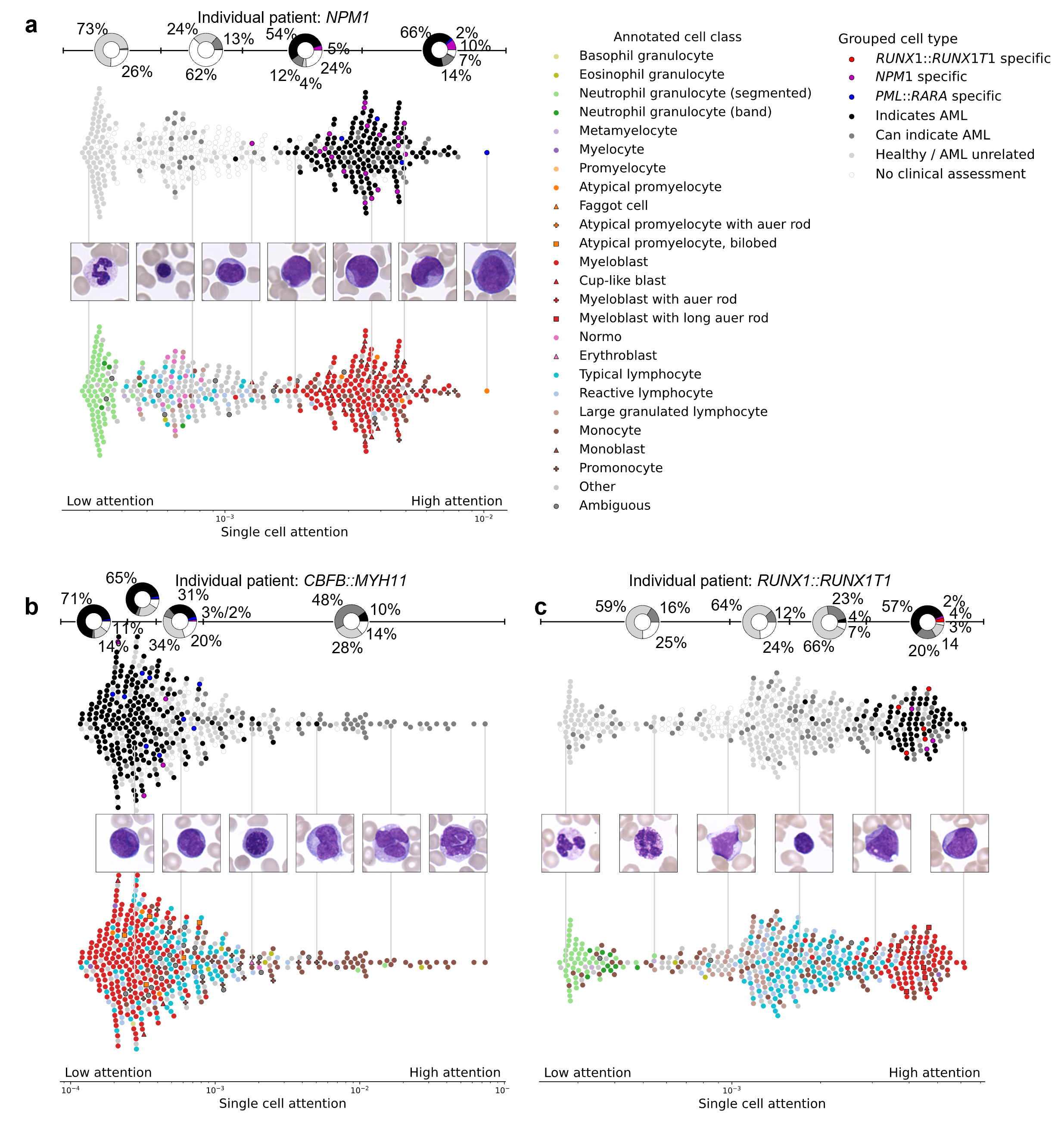

Supplement: S4 Fig — Patients with (a) NPM1, (b) CBFB::MYH11 and (c) RUNX1::RUNX1T1 are classified according to myeloblasts (for CBFB::MYH11: monocytes) present in their corresponding smear. Interestingly, the classification for CBFB::MYH11 (b) mainly focuses on monocytic cells to discriminate this subtype from the other types of AML, while classical myeloblasts receive low attention. Ticks and pie charts at the top indicate quartile ranges and cell group distribution within quartiles. (TIF) [file pdig.0000187.s005.tif]

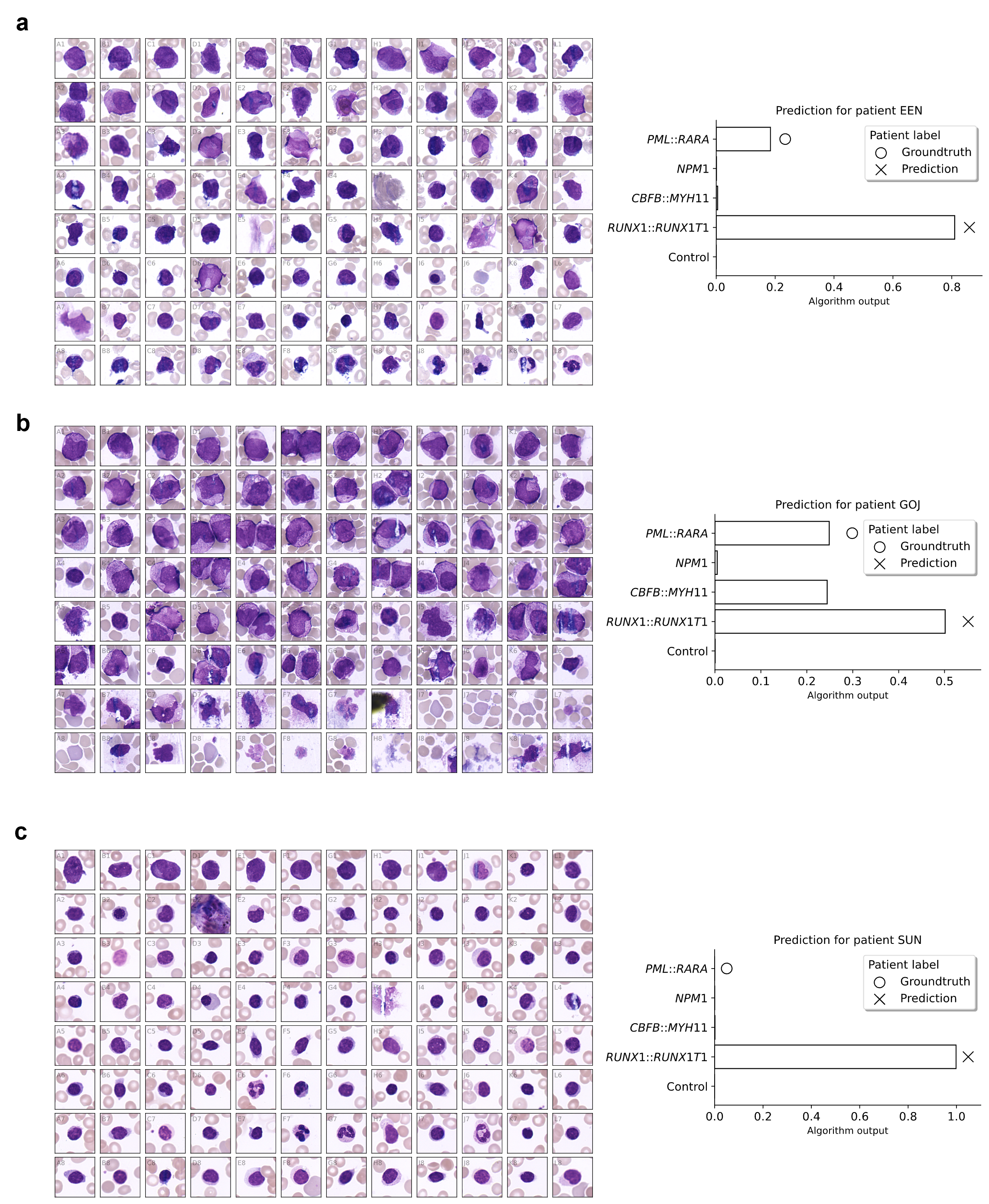

Supplement: S5 Fig — Out of 24 PML::RARA cases in our dataset, 3 cases have been misclassified by SCEMILA (see Fig 2A and Fig 3B) as RUNX1::RUNX1T1. We show 96 representative single-cell images ordered by decreasing attention and the output activation of SCIMILA. (a) Patient EEN contains many white blood cells without intact cytoplasm. Some cells present a bilobed nucleus or stronger granulation. (b) Patient GOJ shows large cells with cytoplasmic granulation as well as some Auer rods. Yet, this patient presents with many artifacts and a lot of red blood cells, some images even contain no white blood cells at all (bottom). Overall the algorithm shows activation for PML::RARA, CBFB::MYH11 and RUNX1::RUNX1T1, indicating uncertainty of the classification. (c) While the fraction of neutrophil granulocytes is quite small, patient SUN presents with few suspicious PML-RARA cells. (TIF) [file pdig.0000187.s006.tif]

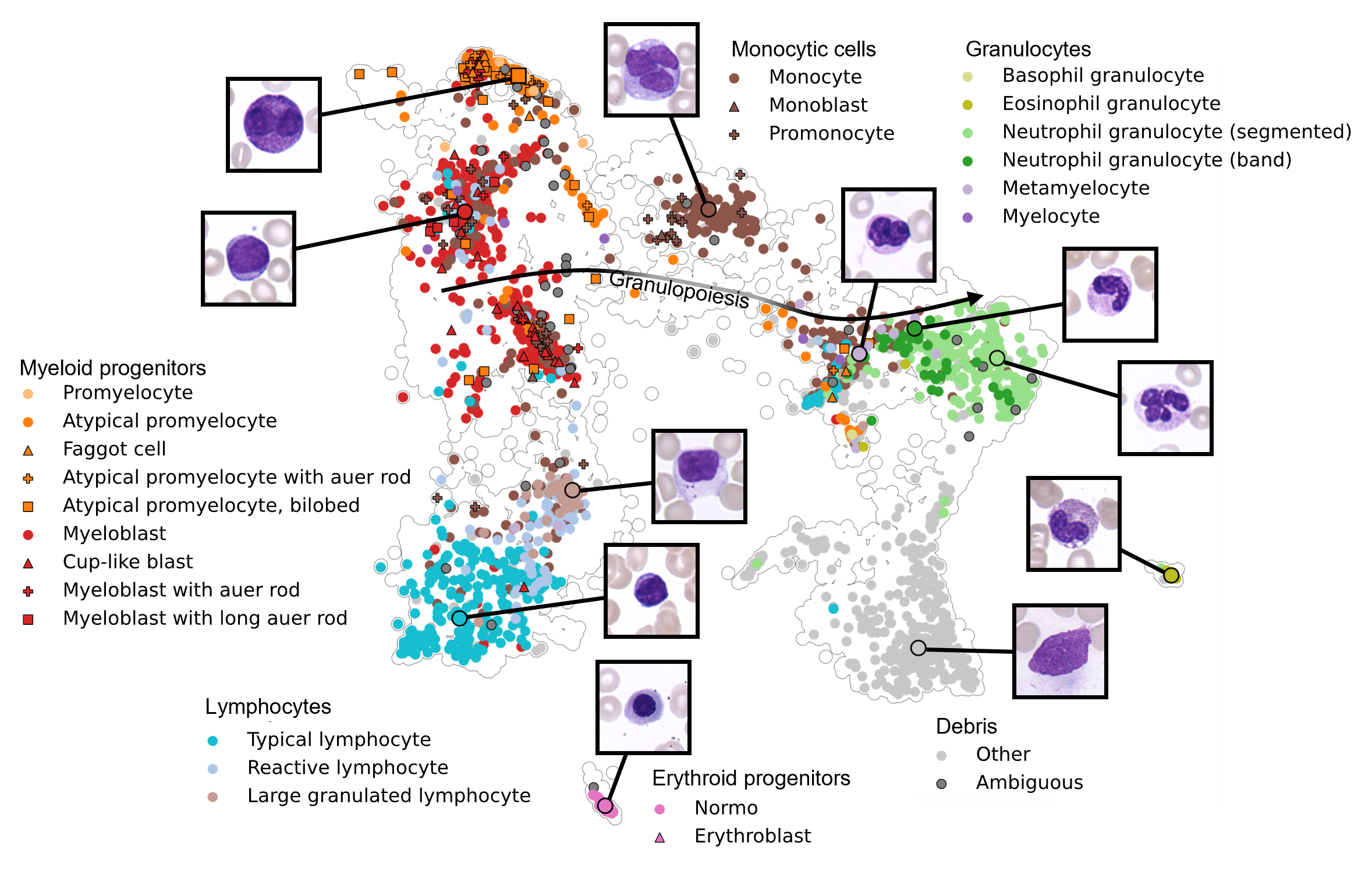

Supplement: S6 Fig — All single-cell images from one fold were embedded based on extracted features, using the uniform manifold approximation and projection method (UMAP), and are abstracted by a gray contour. 1983 cells from 4 patients, annotated by an expert hematologist after training, are highlighted, including cells specific for different genetic subtypes of AML. Images show exemplary single cells, clusters for debris (gray), neutrophil granulocytes (green), myeloblasts (red) and (atypical) promyelocytes (orange), lymphocytes (blue), monocytes (brown) were manually annotated. The black arrow highlights the differentiation trajectory from myeloblasts over promyelocytes, myelocytes, metamyelocytes and band neutrophil granulocytes to segmented neutrophil granulocytes. (TIF) [file pdig.0000187.s007.tif]

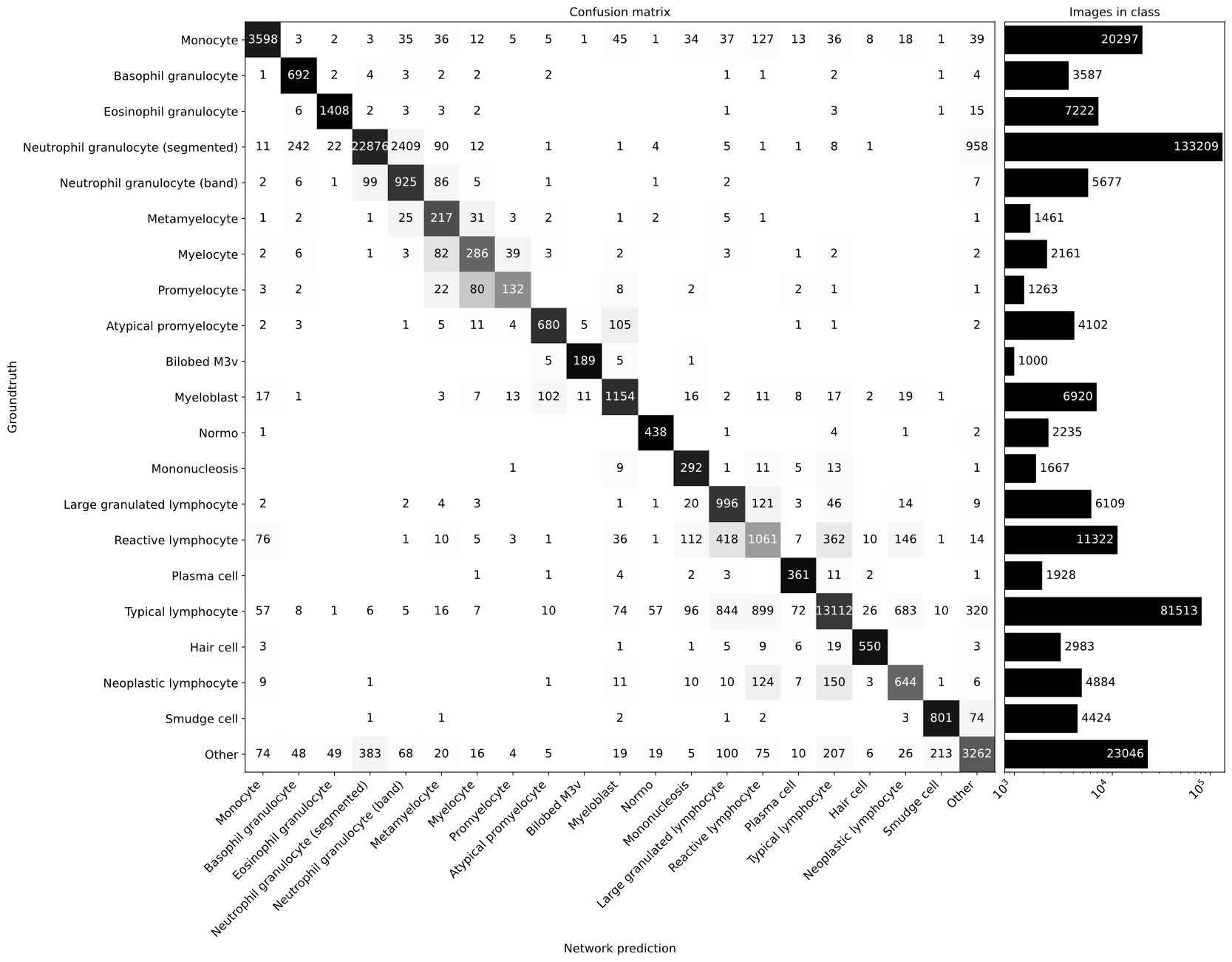

Supplement: S7 Fig — (TIF) [file pdig.0000187.s008.tif]
